# Statistical efficiency and optimal design for stepped cluster studies under linear mixed effects models

**DOI:** 10.1002/sim.6850

**Published:** 2016-01-07

**Authors:** Alan J. Girling, Karla Hemming

**Affiliations:** ^1^ Institute of Applied Health ResearchUniversity of BirminghamB15 2TTU.K.

**Keywords:** cluster studies, stepped‐wedge designs, intra‐cluster correlation, optimal design

## Abstract

In stepped cluster designs the intervention is introduced into some (or all) clusters at different times and persists until the end of the study. Instances include traditional parallel cluster designs and the more recent stepped‐wedge designs. We consider the precision offered by such designs under mixed‐effects models with fixed time and random subject and cluster effects (including interactions with time), and explore the optimal choice of uptake times. The results apply both to cross‐sectional studies where new subjects are observed at each time‐point, and longitudinal studies with repeat observations on the same subjects.

The efficiency of the design is expressed in terms of a ‘cluster‐mean correlation’ which carries information about the dependency‐structure of the data, and two design coefficients which reflect the pattern of uptake‐times. In cross‐sectional studies the cluster‐mean correlation combines information about the cluster‐size and the intra‐cluster correlation coefficient. A formula is given for the ‘design effect’ in both cross‐sectional and longitudinal studies.

An algorithm for optimising the choice of uptake times is described and specific results obtained for the best balanced stepped designs. In large studies we show that the best design is a hybrid mixture of parallel and stepped‐wedge components, with the proportion of stepped wedge clusters equal to the cluster‐mean correlation. The impact of prior uncertainty in the cluster‐mean correlation is considered by simulation. Some specific hybrid designs are proposed for consideration when the cluster‐mean correlation cannot be reliably estimated, using a minimax principle to ensure acceptable performance across the whole range of unknown values. © 2016 The Authors. Statistics in Medicine published by John Wiley & Sons Ltd.

## 1 Introduction

**Figure 1:**
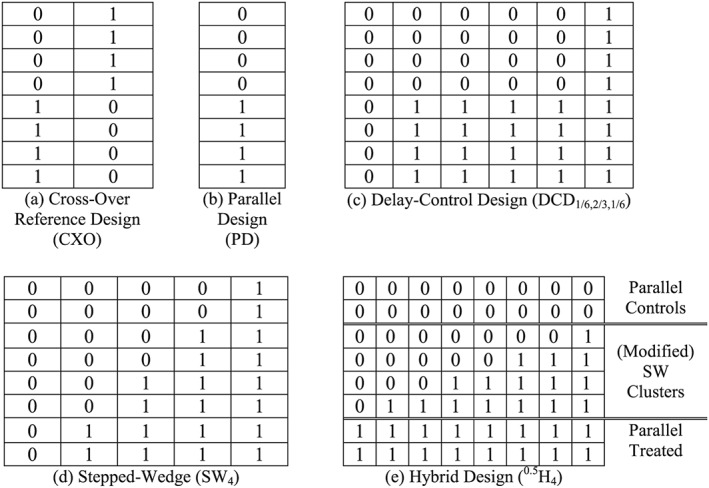
Schematic for some designs with eight clusters. The horizontal direction represents time and the total duration of the study (which is the same for each design) is equally divided between the columns. The rows represent clusters. So each cell represents the observation‐times in a single cluster over one fixed time period. Treated cells are denoted by 1, and Controls by 0.

In a ‘stepped’ cluster design, an intervention is introduced into some or all of the clusters at (possibly) different uptake‐times during the study. Once introduced into a cluster, the intervention persists until the end of the study. We assume that outcome‐data is collected in all clusters throughout the duration of study and that interest centres on the contrast between the treated (post‐intervention) and control (pre‐intervention) conditions. This class of study design includes some traditional parallel cluster designs 1 with ongoing patient recruitment (where one group of clusters receives the intervention at the start, and the remainder at the end, or not at all) as well as the recent ‘stepped‐wedge’ designs in which the intervention is introduced into all clusters but at staggered (often regularly spaced) time‐points 2, 3, 4, 5. Some other examples feature in Figure 1. Stepped‐wedge designs offer a potential advantage over parallel designs in that each cluster functions as its own control. This can be useful when a high proportion of the variation occurs at the cluster level, but it must be set against the temporal confounding that may arise because the number of clusters exposed to the intervention increases over the course of the study. The tension between cluster‐level effects and temporal confounding is a critical factor for the performance of stepped designs 6.

We suppose that a study for a cluster‐level intervention is planned to take place in *K* clusters and that each cluster contributes *m* outcome measurements (observations) at each of *T* regularly‐spaced time‐points. The discussion here applies to two main types of cluster study: (i) longitudinal (cohort) studies where *Km* subjects are recruited at the beginning of the study, with repeat observations at each time‐point; and (ii) cross‐sectional studies where *KTm* different subjects are recruited during the course of the study, and each contributes a single observation on the main outcome measure. We exclude ‘open’ cohort designs – i.e. longitudinal studies with significant drop‐out and/or ongoing recruitment. Instances of these two types are provided by two paradigms: (i) studies of a health intervention in a closed population (such as a care‐home) where the same subjects are monitored over a period of time 7, 8; and (ii) service‐delivery studies in hospital in‐patient or emergency departments where a single outcome measure is obtained from each member of a continuously changing patient population 9, 10. Further instances of each type are enumerated in a recent review 4. In either case we assume that the frequency and timing of data‐collection are already settled, and focus on the choice of intervention uptake‐times: specifically the impact of this choice on the statistical performance of the intervention effect estimate.

The observations are described by a linear mixed effects model with fixed time and random cluster effects due to Hussey and Hughes 11, with the addition of random cluster‐specific time components and subject‐level components for longitudinal studies 12. Even without these additions, the Hussey and Hughes model has been found useful for the design of stepped studies 13, 14. It is directly applicable when the outcome is continuous, and approximately for binary outcomes when *m* is large 11. It was used also in a recent paper that addresses the optimal design of stepped‐wedge studies 15, although under conditions that exclude most of the optimal designs in the present study.

Some designs using eight clusters are illustrated in Figure 1. In each particular design the number of observation‐times in each cell is constant, with treatment status coded as 1 if exposed and 0 if unexposed to the intervention. The total number of observations in each cluster (*mT* = *M*) is the same for all designs. This means that the cell‐sizes may differ between designs. Thus a cell in Figure 1b – the parallel design – contains twice as many observations as a cell in Figure 1a.

In most of these examples the number of treated clusters grows over the course of the study. An exception is the cross‐over design in Figure 1(a). This design is often not practical because the intervention is withdrawn in some clusters halfway through the study. However it is useful as a reference design against which the relative efficiency of other designs can be calibrated. The ‘Delay‐Control Design’ – a terminology suggested by an anonymous referee – in Figure 1c is an elaboration of the parallel design in which all clusters contribute baseline control observations and all receive the intervention towards the end of the study. A stepped‐wedge design arises if all clusters receive the intervention with uptake times evenly spaced throughout the study. The stepped‐wedge in Figure 1d has eight clusters but only g = 4 uptake times. Alternatives with g = 2 or 8 are also available. The final design (Figure 1e) is motivated by an optimality result described later. It represents a 50:50 hybrid combination of a parallel design in the top and bottom two clusters with a stepped‐wedge‐type layout in the middle four clusters, although with reduced time before the first and after the last uptake point.

The paper contains two main sections. In section 2 a generic expression for the precision of the treatment effect estimate is obtained. This leads to a simple graphical method for comparing the efficiency of designs. Section 3 is concerned with the optimal choice of uptake times when the numbers of clusters and observation time‐points are both fixed, and includes some suggestions for practical design choices.

## 2 The precision of the effect estimate under a linear mixed effects model

### 2.1 The model

Each cluster *i* (*i* = 1,…,*K*) generates *m* measurements at every time‐point *j* (*j* = 1,…,*T*) according to the following model:

(1) $$Y_{ijl}^{\prime }=c_{i}+t_{j}+\theta J_{ij}+{\left(ct\right)}_{ij}+s_{l\left(i\right)}+{\left(st\right)}_{l\left(i\right)j}\text{.}$$

Here $Y_{ijl}^{\prime }$ has variance $\sigma_{1}^{2}$ and is the outcome for the *l*th observation (*l* = 1,…,*m*) at time *j* in cluster *i*; *c*_1_,…,*c_K_* are mutually independent random cluster effects (variance $=\eta_{C}\sigma_{1}^{2}$); *t*_1_,…,*t_T_* are fixed time‐effects; *s*_*l*(*i*)_ and (*st*)_*l*(*i*)*j*_ are within‐cluster random effects with variances $\eta_{S}\sigma_{1}^{2}$ and $\eta_{ST}\sigma_{1}^{2}$ respectively; (*ct*)*_ij_* represents cluster‐level fluctuations of the time‐effect with variance $\eta_{CT}\sigma_{1}^{2}$ so that *η*_*CT*_ = 1 − *η*_*C*_ − *η*_*S*_ − *η*_*ST*_; *θ* is the intervention effect whose estimation is the purpose of the analysis; and *J_ij_* is a binary variable (as in Figure 1) which indicates whether or not the intervention is present at time *j* in cluster *i*. The random components in this model are as in Teerenstra *et al*. 12. Note that we use *M* = *Tm* to denote the total number of observations in each cluster. The number of time parameters is dictated by the number of observation times (*T*), and this may be greater than the number of columns in the representation used in Figure 1. However, the main precision formula below applies also for models with a single time‐parameter for each column.

This formulation covers two important cases:

1. Cross‐sectional study with no within‐cluster time effects: *η*_*S*_ = 0, *η*_*CT*_ = 0. At each time‐point *m* (new) subjects are observed within each cluster. The (*st*) component combines variation between subjects (within clusters) and within‐subject measurement error. Here *η_C_* is the conventional intra‐cluster correlation coefficient (ICC).
2. Longitudinal/Cohort study: *η*_*S*_ > 0. Within each cluster, the same group of *m* subjects is measured at every time‐point. The *s*‐component describes variation between subjects (within clusters); the (*st*)‐component describes variation within‐subjects (including measurement error); the (*ct*)‐component describes cluster‐level time effects.

In this work we consider the performance of the best linear unbiased estimate of *θ* in model (1). This can depend only on the means of the observations at each time‐point in each cluster, i.e. on $Y_{ij}={\bar{Y^{\prime }}}_{ij•}=\frac{1}{m}\sum_{l=1}^{m}Y_{ijl}^{\prime }$. The *Y_ij_*s obey a model (2) of the form proposed by Hussey and Hughes 11 and which is the basis of our development. This is:

(2) $$Y_{ij}=\gamma_{i}+t_{j}+\theta J_{ij}+\varepsilon_{ij};\;1\leq i\leq K,\;1\leq j\leq T\text{,}$$

where $\gamma_{i}=c_{i}+{\bar{s}}_{•\left(i\right)}$, with variance $\left(\eta_{C}+\frac{\eta_{S}}{m}\right)\sigma_{1}^{2}$, and $\varepsilon_{ij}={\left(ct\right)}_{ij}+{\bar{\left(st\right)}}_{•\left(i\right)j}$, with variance $\left(\eta_{CT}+\frac{\eta_{ST}}{m}\right)\sigma_{1}^{2}$, are independent random components. The variance of *Y_ij_* can be partitioned as

$$\mathrm{var}Y_{ij}=\sigma^{2}=\left(\eta_{C}+\frac{\eta_{S}}{m}+\eta_{CT}+\frac{\eta_{ST}}{m}\right)\sigma_{1}^{2}=\mathrm{var}\gamma_{i}+\mathrm{var}\varepsilon_{ij}=\rho \sigma^{2}+\left(1-\rho \right)\sigma^{2}$$

with $\frac{\rho }{1-\rho }=\frac{m\eta_{C}+\eta_{S}}{m\eta_{CT}+\eta_{ST}}$. The quantity *ρ* is the correlation between *Y_ij_*‐values obtained in the same cluster at different times, and corresponds to the ICC for the *Y_ij_*s. Unlike ICCs for the individual observations $Y_{ijl}^{\prime }$, ρ can easily be large (i.e. close to 1), particularly if *η_CT_* = 0.

### 2.2 The cluster‐mean correlation

The efficiency of a stepped cluster design turns on the quantity *R*, defined by $R\left(T,\rho \right)=\frac{T\rho }{1+\left(T-1\right)\rho }$, from which $\frac{R}{1-R}=T\frac{\rho }{1-\rho }=T\frac{m\eta_{C}+\eta_{S}}{m\eta_{CT}+\eta_{ST}}$. The value of *R* has a simple interpretation in terms of the *Y_ij_* as the proportion of the variance of a cluster‐mean ${\bar{Y}}_{i•}\left(={\bar{t}}_{•}+\gamma_{i}+{\bar{\varepsilon }}_{i•}\right)$ that comes from random effects that are independent of time (i.e. from the *c_i_* and *s_l(i)_*). In many circumstances this definition is aligned with that for the correlation between the means of two replicate sets of observations – say ${\bar{Y}}_{i•}^{\left(1\right)},{\bar{Y}}_{i•}^{\left(2\right)}$ – from the same cluster *i*, although the nature of the replication must be carefully considered. For a cross‐sectional study with *η_S_* = 0, *η_CT_* = 0 the replicate set simply entails the recruitment of a new set of subjects in the same cluster. Moreover, in this case, $R\left(T,\rho \right)=R\left(M,\eta_{C}\right)=\frac{M\eta_{C}}{1+\left(M-1\right)\eta_{C}}$, furnishing a direct connection with *η_C_*, the ICC for the individual observations. A similar interpretation in longitudinal studies is possible if the ‘replicate set’ is taken to require new observations at the same times on the same subjects rather than a set of measurements on new subjects.

**Figure 2:**
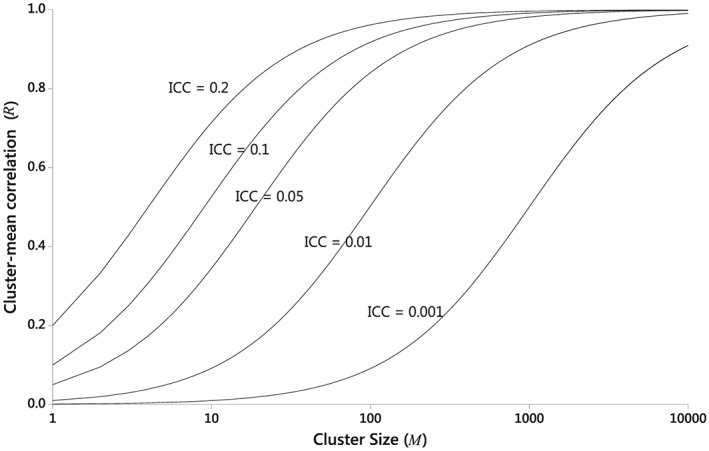
The cluster‐mean correlation (*R*) as a function of cluster‐size (*M = Tm*) in cross‐sectional studies with individual‐observation ICC (*η_C_*) = 0.001, 0.01, 0.05, 0.10, 0.20.

In all cases we refer to *R* as the cluster‐mean correlation (CMC). In general 0 ≤ *R* ≤ 1 and large values of *R* (i.e. close to 1) are possible even in cross‐sectional studies with a small ICC (*η_C_*) for the individual observations. This feature is illustrated in Figure 2.

### 2.3 A precision formula

The performance of the design can be identified with the precision of the best linear unbiased estimate (BLUE) of the treatment effect *θ* in model (1), with known variances. The BLUE under model (1) is also the BLUE under the Hussey and Hughes model (2), so that the following result can be applied:

(*Precision of the effect estimate*) Under the model in (2) the BLUE of θ has precision given by:

(3) $$\Pi ={\left(\mathrm{var}\hat{\theta }\right)}^{-1}=\frac{KT}{\sigma^{2}\left(1-\rho \right)}\times \left(a_{D}-b_{D}R\left(T,\rho \right)\right)$$

where $R\left(T,\rho \right)\text{≡}\frac{T\rho }{1+\left(T-1\right)\rho }$ is the CMC and *a_D_* and *b_D_* are constants, specific to the particular design *D*, that depend on the distribution of the treatment indicator *J_ij_* over the units in the study.

Specifically we have

$$a_{D}=\frac{1}{TK}\sum_{j=1}^{T}\sum_{i=1}^{K}{\left(J_{ij}-{\bar{J}}_{•j}\right)}^{2}$$

and

$$b_{D}=\frac{1}{K}\sum_{i=1}^{K}{\left({\bar{J}}_{i•}-{\bar{J}}_{••}\right)}^{2}\text{,}$$

making use of the ‘dot‐bar’ notation, so that ${\bar{J}}_{i•}$ means $\frac{1}{T}\sum_{j=1}^{T}J_{ij}$ and ${\bar{J}}_{••}$ means $\frac{1}{TK}\sum_{j=1}^{T}\sum_{i=1}^{K}J_{ij}$.

In the language of the analysis of variance, *a_D_* is the ‘within‐columns’ variance of the binary variable *J*_*ij*,_ and *b_D_* is the ‘between‐rows’ variance of *J_ij_*, under a probability model in which equal weight is assigned to each point of the (*i,j*) lattice. The result (3) is established in Appendix A under the conditions of model (2), and provides a straightforward method to calculate the precision for any configuration of *J_ij_*s.

The argument in appendix also shows that the precision expression (3) holds for a model with linear constraints on the *t_j_* parameters, provided that the resulting model is rich enough to represent an initial level, together with a separate time‐effect for each uptake point. If the design is represented as in Figure 1, the minimum requirement is a separate time parameter for each column.

(*Extension to fixed cluster effects*). If the cluster effects (γ*_i_*) are treated as fixed, the precision of the estimate of *θ* is recovered by setting *R*(*T,ρ*) = 1 in expression (3).

The result (3) is formally equivalent to an expression given by Hussey and Hughes 11. However, these authors do not expose the important relationship with the CMC.

### 2.4 Precision for some particular designs

The design coefficients *a_D_* and *b_D_* in (3) are presented for some particular designs in Table 1 below. The calculations are outlined in Appendix B. In some cases these expressions apply strictly only if certain divisibility conditions on *T* and *K* are met. For example, the results for the *g*‐step stepped‐wedge design require that *T* be a multiple of (*g* + 1), and *K* a multiple of *g*. Where a required condition does not hold, the results may be regarded as approximations whose validity improves in larger studies.

**Table 1:** Design coefficients for some selected designs. These determine the precision using equation (3) and the design effect using equation (5). The final column represents the asymptotic relative efficiency (ARE) of the design (compared to CXO) when the number of observations is large.

Design (*D*)	Example	4*a_D_*	4*b_D_*	ARE (*T* → ∞) = 4*a_D_* − 4*b_D_*
Cross‐Over (CXO)	Figure 1a	1	0	1
Parallel Design (PD)	Figure 1b	1	1	0
Delay‐Control Design (DCD_*p*,*q*,*r*_)	Figure 1c	*q*	*q^2^*	*q*(1 − *q*)
Stepped‐Wedge (SW*_g_*)	Figure 1d	$\frac{2}{3}\left(1-\frac{1}{g}\right)$	$\frac{1}{3}\left(1-\frac{2}{g+1}\right)$	$\frac{1}{3}\left(1-\frac{2}{g\left(g+1\right)}\right)$
Modified Stepped‐Wedge (MSW*_g_*)	Figure 1e (middle 4 clusters)	$\frac{2}{3}\left(1-\frac{1}{g^{2}}\right)$	$\frac{1}{3}\left(1-\frac{1}{g^{2}}\right)$	$\frac{1}{3}\left(1-\frac{1}{g^{2}}\right)$
Hybrid (^β^H*_g_*)	Figure 1e	$1-\frac{\beta^{2}}{3}\left(1+\frac{2}{g^{2}}\right)$	$1-\frac{\beta }{3}\left(2+\frac{1}{g^{2}}\right)$	$\frac{\beta }{3}\left\{2+\frac{1}{g^{2}}-\beta \left(1+\frac{2}{g^{2}}\right)\right\}$

Cluster Cross‐Over design (CXO): For this design it is assumed that both *K* and *T* are even. Initially there are *K*/2 intervention clusters and *K*/2 control clusters. Halfway through the study each cluster crosses over to the alternative condition.

Parallel Design (PD): This is a classic design for a cluster‐randomised trial. An even number of clusters is assumed, equally divided between treated and control conditions.

Delay‐Control Design (DCD_*p*,*q*,*r*_): A parallel layout runs only for a proportion *q* of the study duration, preceded by a period (proportion *p*) in which all clusters remain in the control condition, and followed by a period (proportion *r*) in which the treatment is present in all clusters (*p* + *q* + *r* = 1). The PD (= DCD_0,1,0_) and the ANCOVA design 12 (= DCD_0.5,0.5,0_) are covered as special cases. The design coefficients (*a_D_,b_D_)* depend only on the proportion of time (*q*) occupied by the parallel layout.

For both the PD and the DCD it is possible to vary the proportion of control clusters within the parallel layout from the 50% assumed here. The effect would be to reduce both design coefficients – and hence the precision – by the factor 4*s*(1 − *s*) ≤ 1 where *s* is the (new) proportion of control clusters.

Stepped‐Wedge design with g steps (SW_g_): This is the ‘standard’ stepped‐wedge design. The clusters are divided into g equal groups. Initially all clusters are untreated. The uptake time is the same for all clusters in any one group. In the first group this occurs when a fraction of the total study time equal to 1/(*g* + 1) has elapsed and *T*/(*g* + 1) observation time‐points have passed. The uptake times in the remaining groups occur at time‐fractions 2/(*g* + 1), 3/(*g* + 1),…, etc. until all clusters are exposed. During the final part of the study (from time‐fraction *g*/(g + 1) to the end) all clusters are exposed to the intervention.

Modified SW design (MSW*_g_*): In this modification of the SW*_g_* design the time period before the first (and after the last) uptake point is equal to one half of the time between consecutive uptake points – as in the middle four clusters in Figure 1e. Because *a*_*MSW*_ − *b*_*MSW*_ *R* > *a*_*SW*_ − *b*_*SW*_ *R* for all values of *R*, the MSW*_g_* design generates greater precision than the conventional SW*_g_* design, although the relative advantage diminishes as *g* increases.

Stepped‐Wedge/Parallel Hybrid designs (^β^H*_g_*): These designs achieve the maximum precision over stepped designs with overall balance between treated and control observations, a result established below in section 3. In the ^β^H*_g_* hybrid, *K*β clusters are assigned to a modified stepped‐wedge layout with *g* uptake points, and the remaining *K*(1 − β) clusters to a concurrent parallel layout.

### 2.5 Precision‐ratio plots

Using the CXO as a reference design, the efficiency of any stepped design, *D*, may be defined as a precision ratio from (3):

(4) $$\text{Relative Efficiency of design}\;D,\;\frac{\Pi_{D}}{\Pi_{CXO}}=4\left(a_{D}-b_{D}R\left(T,\rho \right)\right)\text{.}$$

The performance of the design when *T* is large is captured by the asymptotic relative efficiency (ARE) as *R* → 1 (= 4*a_D_* − 4*b_D_*) as given in the final column of Table 1. The fact that this is zero for the PD reflects the generally poor performance of parallel cluster trials with large cluster sizes 16. The other designs all have non‐zero AREs so that there is no upper limit to the precision that can be obtained by increasing the number of observations in each cluster.

**Figure 3:**
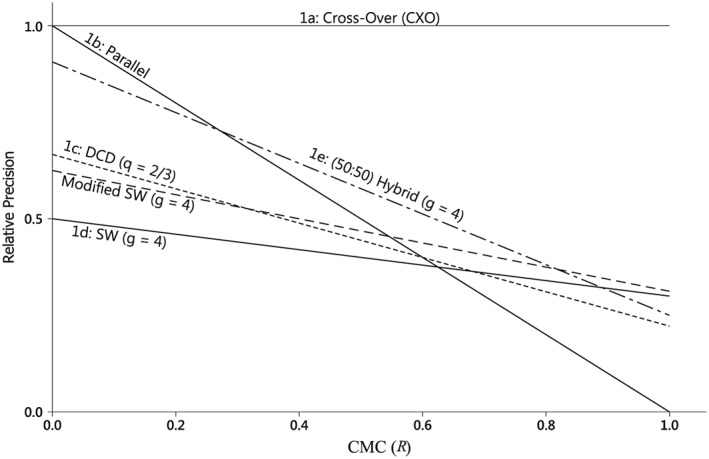
Precision–ratio plots. The relative precision of some stepped designs compared to the cross‐over design is plotted against the cluster‐mean correlation parameter *R*. Designs 1a to 1e are those illustrated for eight clusters in Figure 1. The Modified SW design corresponds to two copies of the middle four clusters in Figure 1e.

Figure 3 shows the precision‐ratio as a function of the CMC, *R*, for the designs in Figure 1. When *R* is small, the PD outperforms the other designs, but is the worst design when *R* = 1. The SW_4_ design performs relatively well for large *R* but is dominated by MSW_4_. Similarly DCD with *q* = 2/3 is dominated by ^0.5^H_4_. Over the range 311≤R≤911 the ^0.5^H_4_ design gives the best precision among these designs. Outside this range PD (for smaller *R*) or MSW_4_ (larger *R*) are best. If the value of *R* is uncertain the ^0.5^H_4_ design might be preferred as a compromise design, at least on statistical grounds, because it performs reasonably well throughout the range.

This approach can inform the choice between any two candidate designs: for example, that between a stepped‐wedge and parallel design 14, 16, 17, 18, 19, 20, 21, 22. From (4) the stepped‐wedge is the more efficient only if *R* > *r*_0_ where the threshold *r*_0_ satisfies

$$a_{SW}-b_{SW}r_{0}=a_{PD}-b_{PD}r_{0}\text{.}$$

This implies that $r_{0}=\frac{1}{2}\left(1+\frac{1}{g}\right)$ for a standard SW_g_ design, and $r_{0}=\frac{1}{2}\left(1+\frac{3}{1+2g^{2}}\right)$ if an MSW*_g_* is used. In general, a SW design – of either type – can be better than a PD only if the cluster‐mean correlation *R* exceeds ½. In a cross‐sectional study, where the ICC of a single observation (i.e. *η_C_*) is generally small, this translates into a useful rule of thumb: to prefer the SW option only if the ICC is greater than 1/*M*.

### 2.6 Design effects

A design effect is the ratio of the precision of the treatment effect estimate in an individually randomised trial (RCT) to that in the cluster trial, and is convenient for sample size calculations. For a CXO design under model (1) the components *c_i_* and *s_l(i)_* cancel out in the treatment‐effect estimate and the design effect is just *mη*_*CT*_ + *η*_*ST*_. It follows that the design effect for a general design *D* is given by

(5) $$\text{Design Effect}\;=\left(m\eta_{CT}+\eta_{ST}\right)\times \frac{\Pi_{CXO}}{\Pi_{D}}=\frac{m\eta_{CT}+\eta_{ST}}{4\left(a_{D}-b_{D}R\right)}\text{.}$$

For a parallel cross‐sectional (PD) design with *M* observations per cluster and no cluster‐level time effects (*η_S_* = *η_CT_* = 0) the formula (5) becomes (1 − *η*_*C*_)/(1 − *R*) and yields the well‐known design effect [1 + (*M* − 1)*η*_*C*_] 1. Published stepped‐wedge design effects 14 can also be obtained in this way.

## 3 Finding the best design

In many traditional designs the intervention is introduced (into some of the clusters) at a single time‐point in the study. This is not true of stepped‐wedge designs for which a number (*g*) of different uptake times will be required. Normally these are equally spaced in time, yet they can be chosen so as to optimise the performance of the design. To this end, we assume that the study will involve *T* observation times within each of *K* clusters with observations *Y_ij_* generated by the model (2). The problem is to choose a configuration of uptake times in the clusters to achieve the best possible precision for the treatment effect estimate. Note that it is possible for an uptake time to occur before the start of the study (in a pure ‘treated’ cluster), or, notionally, after it has finished (in a pure ‘control’ cluster).

Mathematically we must find the values of the *J_ij_*s (= 0 or 1) that maximise the expression for Π in (3), subject to the irreversibility constraint that *j* > *j*′ ⇒ *J_ij_* ≥ *J*_*ij*′_ for all *i*. First suppose that the clusters have been numbered according to the order in which their uptake times occur (so that *i* < *i′* ⇒ *J_ij_* ≥ *J_i′j_* for all *j*). This ordering has been implicitly assumed in the examples in Figure 1 above, with the cluster index *i* taken as increasing in the upwards direction and the time index *j* as increasing from left to right. Also it is convenient to map the design points (*i*,*j*) on to a lattice in the *x‐y* plane by setting

$$x_{j}=\frac{1}{T}\left(j-\frac{T+1}{2}\right),\;j=1,2,\ldots ,T;\;y_{i}=\frac{1}{K}\left(i-\frac{K+1}{2}\right),\;i=1,2,\ldots ,K\text{.}$$

**Figure 4:**
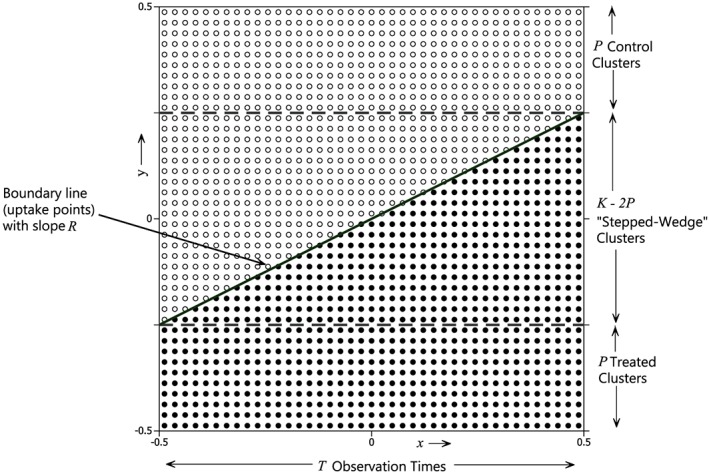
A best balanced design. The boundary line (of slope *R*) divides the treated (●) from the untreated (○) points, and contains the uptake points in the ‘stepped‐wedge’ clusters. If *T*/(*K* − 2*P*) is an even integer, the design is a Hybrid design as defined above in section 2.

The *x–y* design lattice is contained in a unit square centred on the origin (see Figure 4).

With these definitions an alternative expression to (3) for the precision is given by

(6) $$\Pi ={\left(\mathrm{var}\hat{\theta }\right)}^{-1}=\frac{1}{\sigma^{2}\left(1-\rho \right)}\left\{2\sum_{i,j}\left(Rx_{j}-y_{i}\right)J_{ij}-R\left(J_{••}-\frac{1}{TK}J_{••}^{2}\right)\right\}\text{,}$$

a result derived in Appendix C. Unlike (3) (which holds for any configuration of the *J_ij_*s) the expression (6) is valid in the presence of the irreversibility constraint, and where the clusters follow the suggested ordering.

### 3.1 An algorithm for optimal designs

Suppose that *J*_• •_ (the total number of treated time‐points over all clusters) is fixed. Then, in view of (6), the precision is maximised by nominating treated points one by one, beginning with (*i*, *j*) = (1, *T*) and in decreasing order of the quantity (*Rx_j_ – y_i_*), until exactly *J*_• •_ are included in the treated set. Any ambiguity in the ordering caused by tied values can be resolved arbitrarily because tied points contribute the same amount to the expression in (6). In geometrical terms this is achieved by moving a straight line of slope *R* upwards until exactly *J*_• •_ points lie on or beneath it (as, for example, in Figure 4). In this way designs with the best precision are determined for each of the (*TK* − 1) possible values of *J*_• •_. The overall optimum can then be obtained by maximising the best precision over *J*_• •_, using (6). This algorithm is easily implemented in a spreadsheet programme and can assist with the design of practical studies.

### 3.2 Best balanced designs

Practical experience with this algorithm shows: (i) that the optimal design is not always unique; and (ii) that when the number of design points is an even number there may still be no optimal design with overall balance between the treatment arms – i.e. for which $J_{••}=\frac{1}{2}TK$. Notwithstanding (ii) it turns out that the design with greatest precision among those that satisfy the balance condition (the best balanced design, or BBD) is often approximately optimal, especially when both *T* and *K* are large.

The BBD can be determined from the algorithm above by setting $J_{••}=\frac{1}{2}TK$.

(*Best Balanced Design*) Suppose that the number of design points, *TK*, is even. A design in which the treated points consist of those for which *y_i_* < *Rx_j_* together with half of the points (if there are any) for which *y_i_* = *Rx_j_* is a best balanced design.

In such a design, the boundary between the treated and untreated lattice‐points is a straight line through the origin with slope *R*. An example is shown in Figure 4. The first group of *P* clusters receive the intervention at the start of the study (i.e. they are ‘Treated’ clusters) where *P* is the label of the last cluster for which the point (*x*_1_ *,y_P_*) lies on, or just below, the boundary line *y* = *Rx*. This condition translates into the inequalities:

$$KR\left(1-\frac{1}{T}\right)-1\leq K-2P\leq KR\left(1-\frac{1}{T}\right)+1\text{.}$$

The last *P* clusters are ‘control clusters’ and do not receive the intervention at all. The *K* − 2*P* clusters in the middle participate in a genuine stepped study with equal intervals of time between the uptake points. In some cases the design for these middle clusters corresponds exactly to a MSW_(*K* − 2*P*)_ design, though this requires that *T* = 2(*K* − 2*P*)*l* for some integer *l*, where *l* is the number of time‐points in each cluster before the first uptake time (and 2*l* is the number between two successive uptake times). In the general case, the correspondence to the MSW design is only approximate.

This result shows that the best balanced design when *T* is large approximates to a hybrid design *^R^*H*_RK_* in which a stepped‐wedge layout is followed in a proportion of the clusters equal to the CMC, *R*, and a parallel layout in the remaining clusters.

### 3.3 Examples of optimal design

Consider the design of a study in 10 clusters over six observation times. The design lattice contains 60 (=10 × 6) points. The observations within each cluster are either unique measurements on 6*m* separate subjects, or they could be six repeat measurements on the same *m* subjects. The value of *m* need not be separately specified because the statistical design issues depend only on the CMC, *R*.

**Figure 5:**
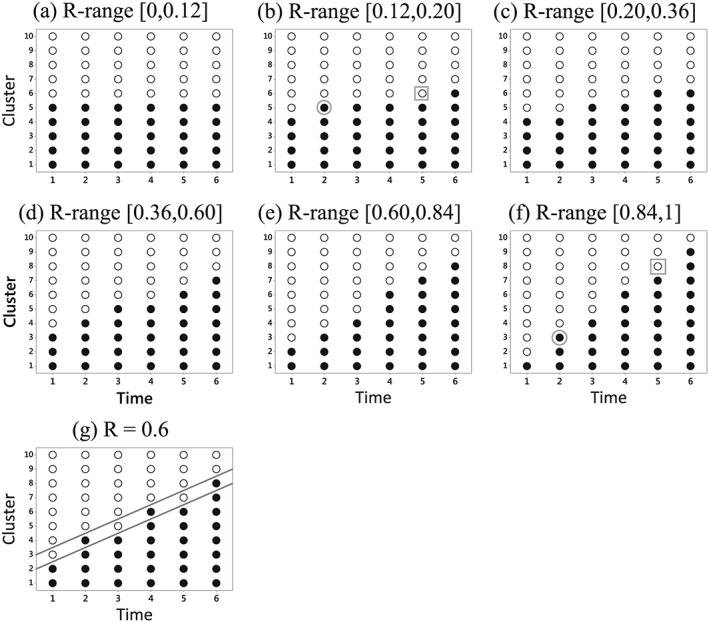
BBDs for 10 clusters over six time‐points. The boxed and circled points in (b) and (f) relate to overall optimal designs for particular *R*‐values as discussed in the text.

Best balanced designs for this problem are shown in Figure 5 for the full range of *R*‐values from 0 to 1. For each design the range of validity includes both end‐points, so that, for instance, design (a) – a parallel layout – and design (b) are both BBDs when *R* = 0.12. The BBDs are unique except at these isolated end‐points. In a majority of cases the BBD is also an optimal design. Exceptions include design (b) for *R* = 0.2, and design (h) for *R* = 1. In each case conversion to optimality is achieved by modifying the treated set either to exclude the circled point or to include the boxed point. By repeated application of the optimality algorithm with R = 0(0.001)1 it was found that the BBD is optimal in 77.5% of cases, and achieved a relative efficiency of at least 98.83% for all values of R, with the worst case at *R* = 0.6. The mean relative efficiency of the BBDs was 99.92% – a minimal loss of efficiency compared to the true optimum.

The case with *R* = 0.6 is complex: designs (d), (e), and (g) are just three out of 20 possible BBDs in this case. The other seventeen BBDs are obtained by choosing different sets of three treated points from the six design points along the diagonal indicated in Figure 5(g), whose slope (=0.6) exactly matches the value of *R*. Design (g) has been singled out here because it is an example of an exact hybrid design (^0.6^H_3_). However, none of these 20 BBDs is fully optimal when *R* = 0.6. An optimal design in this case omits all six diagonal points from the treated set.

All the examples shown include pure ‘control’ clusters and pure ‘treated’ clusters as part of the optimal design. This is a common feature of optimal stepped designs as defined here, but is specifically excluded in recent work by Lawrie *et al*. 15.

### 3.4 Admissible designs for large studies

When *T* and *K* are large, the precision for the best balanced design approximates to that of the overall optimal design, with an error of small order in the quantity min(*T*,*K*). This is shown in Appendix D– by means of an integral approximation to (6). It follows that the efficiency (relative to the CXO) of the overall optimal design for a large study is that of a hybrid design in which *g* is large (i.e. *^R^*H_∞_). From Table 1 this is

(7) $$1-R+\frac{1}{3}R^{2}\text{.}$$

**Figure 6:**
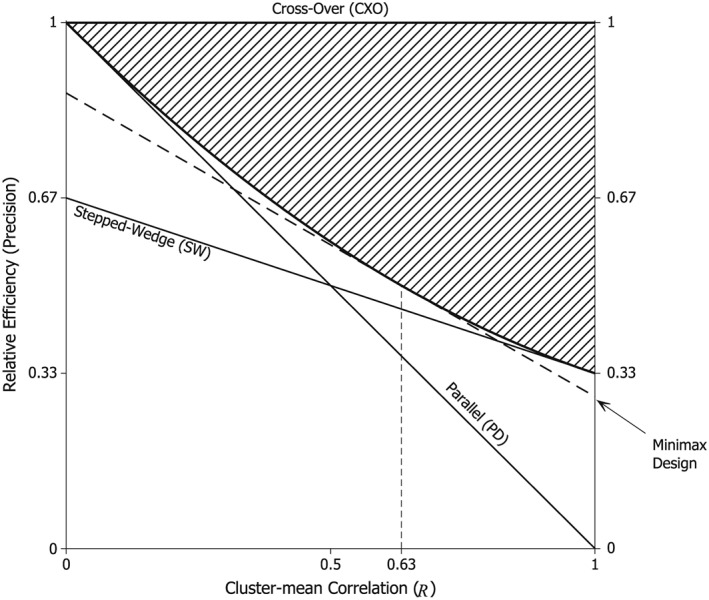
The relative efficiency of cluster designs for large studies as a function of the cluster‐mean correlation *R*. Three feasible (and admissible) stepped designs are shown: PD, SW (solid lines) and Minimax – i.e. ^0.634^H_∞_ – (broken line). The shaded region is prohibited by the irreversibility constraint and bounded by the quadratic curve $1-R+\frac{1}{3}R^{2}$.

This quadratic is plotted in Figure 6 and represents the boundary of the feasible region for stepped designs. The admissible designs (i.e. those that are not dominated by any other stepped design) are the hybrid designs, and generate tangents to the boundary curve. The parallel (*R* = 0) and stepped‐wedge (*R* = 1) designs are both admissible. The region of the unit square in Figure 6 above the quadratic curve is not attainable by any stepped design.

Hybrid designs do not appear to have been widely used in practice. We are aware of only one example 23. However they offer an appealing compromise between the parallel and stepped‐wedge designs given that these can offer optimal precision only at the extreme ends of the range for the cluster‐mean correlation (i.e. when *R* = 0 and 1, respectively). An appropriate hybrid design can improve the precision of the treatment estimate in all other cases.

### 3.5 Minimax designs

Where reliable information about the CMC is not available it makes sense to choose an admissible design that minimises the maximum possible loss of precision relative to the optimal design. For large studies this is a hybrid design ${}^{\tilde{\beta }}H_{\infty }$ (the ‘minimax hybrid’) where $\tilde{\beta }$, the proportion of SW clusters, is given by

$$\tilde{\beta }=\arg\min_{\beta }\max_{R}\left\{1-\frac{1-\frac{1}{3}\beta^{2}-R\left(1-\frac{2}{3}\beta \right)}{1-R+\frac{1}{3}R^{2}}\right\}\text{.}$$

At the minimax solution the relative loss of precision is the same at both ends of the range for *R* (i.e. at *R* = 0 and *R* = 1) because of the convexity of the quadratic boundary curve in Figure 6. So $\tilde{\beta }$ satisfies $\frac{1-\frac{1}{3}{\tilde{\beta }}^{2}}{1}=\frac{1-\frac{1}{3}{\tilde{\beta }}^{2}-\left(1-\frac{2}{3}\tilde{\beta }\right)}{1-1+\frac{1}{3}}$, from which $\tilde{\beta }=\frac{1}{2}\left(3-\sqrt{3}\right)=0.634\ldots$. The precision of the minimax design is at least $1-\frac{1}{3}{\tilde{\beta }}^{2}=\frac{1}{2}\sqrt{3}$ (≈86.6%) of the available precision at all values of *R*.

In practice a near‐minimax design can be constructed if about 63.4% of the clusters are allocated to a stepped‐wedge layout with the remainder in a parallel layout. The extent to which this can be achieved depends on the number of clusters in the study. Ideally, 63.4% of *K* should be close to a whole number of clusters (the SW component), with the residue equal to an even number (for the Parallel component). Some possible near‐minimax designs are shown in Table 2.

**Table 2:** Performance of some near‐minimax hybrid designs alongside minimax (^0.634^H_∞_), PD and SW_∞_ for comparison. The relative precisions are computed with reference to the best possible designs in large studies. When *R* = 0 this is a parallel design (PD), when *R* = 1 it is a MSW with a notionally infinite value of *g*.

Design label	No. of parallel clusters	No. of SW clusters	Total no. of clusters	Proportion of SW clusters (β)	No. of SW uptake points (*g*)	Relative precision at *R* = 0	Relative precision at *R* = 1	Worst relative precision (WRP)
1	2	3	5	0.600	3	85.3	82.7	82.7
2	2	4	6	0.667	4	83.3	87.5	83.3
3	4	6	10	0.600	6	87.3	83.7	83.7
4	4	7	11	0.636	7	86.0	86.4	86.0
5	4	8	12	0.667	8	84.7	88.5	84.7
6	6	9	15	0.600	9	87.7	83.9	83.9
7	6	10	16	0.625	5	85.9	85.3	85.3
8	6	10	16	0.625	10	86.7	85.8	85.8
9	6	12	18	0.667	6	84.4	88.3	84.4
Minimax				0.634	∞	86.6	86.6	86.6
PD				0		100.0	0.0	0.0
SW_∞_				1	∞	66.7	100.0	66.7

**Figure 7:**
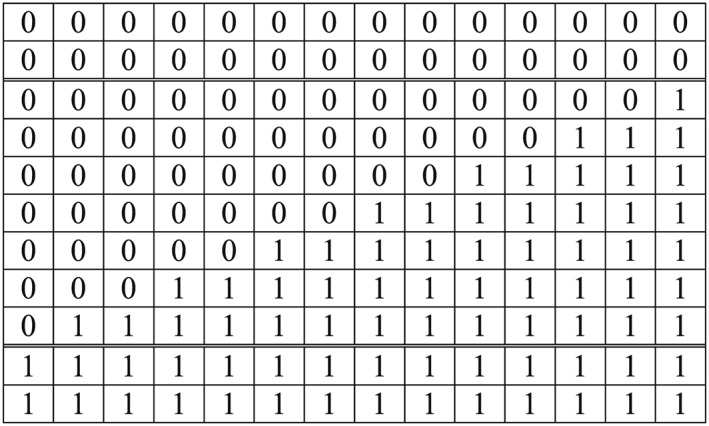
A near‐Minimax design with 11 (groups of) clusters (row 4 from Table 2). It achieves at least 99.25% of the precision of the large‐study minimax design (^0.634^H_∞_) for any value of *R*.

Any of the designs in Table 2 can be ‘scaled up’ to larger numbers of clusters (rows) and observation periods (columns) without the affecting the precision relative to the CXO design, which can depend only on β and *g*. The best performance in the table is achieved by design 4, illustrated in Figure 7. Note that the 50:50 Hybrid in Figure 1e achieves a worst‐case relative precision (WRP) of 75%. Even the simplest five‐cluster design in the table (design 1) achieves a WRP of nearly 83%. This design was encountered above (Figure 5(g)) as a BBD for the case *K* = 10, *T* = 6.

### 3.6 Design performance when *R* is uncertain – a simulation study

In practice the likely performance of a chosen design depends on the degree of uncertainty in the prior estimate of the CMC. This was investigated using a class of prior distributions for *R* of the form:

$$\log\frac{R}{1-R}\sim N\left(\log\frac{R_{0}}{1-R_{0}},\tau^{2}\right)$$

where *R*_0_ is the median CMC value, and prior uncertainty is characterised by the variance parameter *τ* ^2^. Values of τ^2^ were chosen to correspond to three different coefficients of variation of the quantity *R*/(1 − *R*): 0.25 (low uncertainty); 0.5 (medium uncertainty); and 1.0 (high uncertainty). In view of the relation $\log\frac{R}{1-R}=\log T+\log\frac{\rho }{1-\rho }\left[=\;\log M+\log\frac{\eta_{C}}{1-\eta_{C}}\;for\;a\;\text{cross}‐\text{sectional study}\right]$ the prior is consistent with a similar level of uncertainty for *ρ* (or *η_C_*). In particular, this analysis applies directly to the case of a cross‐sectional study with uncertain ICC.

Table 3 shows the results of a simulation study for the distribution of the relative precision of some particular design‐choices compared to the BBD at the true value of *R*. Centiles from the distribution are displayed, alongside the WRP. The designs considered are: (i) BB_0_ – the best balanced design at the median‐estimate, *R*_0_; the near‐minimax hybrid design ^6^H_3_; (iii) Mmx – the true‐minimax design for large studies (= ^0.634^H_∞_). Two cases are shown: (i) a ‘small’ study with 10 clusters and six observation times (for which the BBDs are shown in Figure 5); and (ii) the limiting case of large studies (*T*, *K* → ∞), for which the BBDs are hybrid designs.

**Table 3:** Performance of best balanced and near‐minimax designs under prior uncertainty for the CMC, *R*, (a) for 10 clusters with six observation times and (b) in the limiting case of large studies. The prior median is denoted by *R*_0_, and CV is the coefficient of variation of the quantity *R*/(1 − *R*). the designs considered are: BB_0_ – the BBD at *R*_0_; ^.6^H_3_ – a near‐minimax hybrid design (design 1 in Table 2); Mmx (= ^.634^H_∞_) – the minimax design in large studies. Entries are the centiles (from 9999 simulations) of the relative precision of the stated design compared to the BBD at the true value of *R*. WRP is the relative precision at the least favourable value of *R* (=0 or 1 in all cases).

		(a) *K* = 10, *T* = 6						(b) Limiting case: *T*, *K* → ∞								
		CV = 0.25		CV = 0.5		CV = 1.0		CV = 0.25			CV = 0.5			CV = 1.0		
		BB_0_	^.6^H_3_	BB_0_	^.6^H_3_	BB_0_	^.6^H_3_	BB_0_	^.6^H_3_	Mmx	BB_0_	^.6^H_3_	Mmx	BB_0_	^.6^H_3_	Mmx
*R*_0_ = 0.1	cent50	1.000	0.884	1.000	0.884	1.000	0.884	1.000	0.881	0.895	1.000	0.881	0.895	0.999	0.881	0.895
	cent25	1.000	0.879	0.998	0.876	0.994	0.871	1.000	0.877	0.891	0.999	0.874	0.888	0.998	0.870	0.883
	cent10	0.998	0.876	0.993	0.870	0.978	0.864	1.000	0.874	0.888	0.998	0.869	0.883	0.991	0.864	0.877
	cent5	0.997	0.874	0.990	0.868	0.962	0.861	0.999	0.872	0.886	0.996	0.867	0.880	0.981	0.861	0.874
	cent1	0.994	0.871	0.978	0.864	0.910	0.858	0.998	0.870	0.883	0.991	0.863	0.877	0.944	0.858	0.871
	WRP	0.000	0.853	0.000	0.853	0.000	0.853	0.190	0.827	0.866	0.190	0.827	0.866	0.190	0.827	0.866
*R*_0_ = 0.3	cent50	1.000	0.936	1.000	0.936	0.997	0.936	0.999	0.931	0.949	0.998	0.931	0.949	0.994	0.931	0.949
	cent25	1.000	0.927	0.996	0.921	0.985	0.912	0.998	0.924	0.941	0.994	0.917	0.933	0.985	0.907	0.922
	cent10	0.998	0.921	0.985	0.910	0.959	0.893	0.997	0.917	0.933	0.988	0.905	0.920	0.959	0.889	0.903
	cent5	0.995	0.917	0.977	0.903	0.928	0.884	0.995	0.913	0.929	0.981	0.899	0.914	0.928	0.881	0.895
	cent1	0.988	0.911	0.959	0.893	0.837	0.871	0.991	0.906	0.922	0.960	0.888	0.902	0.853	0.870	0.883
	WRP	0.472	0.853	0.472	0.853	0.472	0.853	0.510	0.827	0.866	0.510	0.827	0.866	0.510	0.827	0.866
*R*_0_ = 0.5	cent50	1.000	0.977	1.000	0.977	0.997	0.971	0.999	0.968	0.990	0.997	0.967	0.989	0.990	0.961	0.984
	cent25	1.000	0.969	0.997	0.963	0.975	0.952	0.997	0.962	0.983	0.990	0.956	0.977	0.973	0.941	0.962
	cent10	1.000	0.963	0.979	0.951	0.932	0.925	0.994	0.956	0.977	0.980	0.944	0.962	0.949	0.919	0.938
	cent5	0.998	0.959	0.963	0.941	0.902	0.913	0.992	0.953	0.973	0.973	0.936	0.954	0.928	0.907	0.925
	cent1	0.985	0.953	0.932	0.924	0.846	0.893	0.987	0.945	0.964	0.954	0.920	0.937	0.881	0.888	0.903
	WRP	0.694	0.853	0.694	0.853	0.694	0.853	0.750	0.827	0.866	0.750	0.827	0.866	0.750	0.827	0.866
*R*_0_ = 0.7	cent50	1.000	0.985	1.000	0.983	1.000	0.975	0.999	0.969	0.997	0.997	0.968	0.995	0.991	0.961	0.988
	cent25	1.000	0.980	1.000	0.973	0.980	0.960	0.998	0.963	0.993	0.991	0.957	0.986	0.976	0.940	0.969
	cent10	1.000	0.974	0.985	0.964	0.951	0.935	0.995	0.957	0.987	0.983	0.942	0.974	0.957	0.914	0.946
	cent5	1.000	0.971	0.972	0.959	0.934	0.921	0.993	0.953	0.984	0.977	0.933	0.965	0.944	0.899	0.932
	cent1	0.989	0.966	0.949	0.941	0.895	0.901	0.989	0.945	0.977	0.963	0.916	0.950	0.915	0.875	0.911
	WRP	0.813	0.853	0.813	0.853	0.813	0.853	0.837	0.827	0.866	0.837	0.827	0.866	0.837	0.827	0.866
*R*_0_ = 0.9	cent50	1.000	0.926	1.000	0.926	1.000	0.926	1.000	0.901	0.936	0.999	0.901	0.936	0.998	0.901	0.936
	cent25	1.000	0.918	1.000	0.911	1.000	0.901	0.999	0.892	0.928	0.998	0.885	0.921	0.995	0.875	0.912
	cent10	1.000	0.911	0.998	0.900	0.979	0.887	0.999	0.885	0.921	0.996	0.873	0.910	0.985	0.857	0.895
	cent5	1.000	0.907	0.991	0.894	0.966	0.880	0.998	0.881	0.918	0.993	0.867	0.904	0.972	0.850	0.888
	cent1	0.999	0.902	0.978	0.887	0.933	0.873	0.997	0.875	0.912	0.984	0.857	0.895	0.933	0.841	0.880
	WRP	0.720	0.853	0.720	0.853	0.720	0.853	0.730	0.827	0.866	0.730	0.827	0.866	0.730	0.827	0.866

The BB_0_ designs show excellent relative precision for medium or low uncertainty about *R*: the median (cent50) relative precision is at least 99% and the 5^th^ centile as least 95% in all cases. Under higher levels of uncertainty there is some deterioration in performance, even in large studies. Even so the 5^th^ centile still exceeds 90% in all tabulated cases. In general, performance is somewhat better for values of *R*_0_ towards the ends rather than the middle of the range.

Nevertheless the low values of the WRP for many of the BB_0_ designs (it can be as low as 0 when *R*_0_ is small) highlight the need for caution if the prior information is unreliable. In this case a more robust choice is the near‐minimax design ^.6^H_3_. In the small study its WRP of 85.3% guarantees acceptable performance even when the CMC is very low. In large studies, it is inferior to the true minimax design (^0.634^H_∞_) by less than 4 percentage points (i.e. 0.04) at every centile of the distribution of relative precision. Improved near‐minimax designs are available in Table 2.

## 4 Comments

The mixed effects model (1) is a vehicle to express a certain correlation‐structure with additive fixed effects for time and treatment, including subject‐level effects and variation within clusters over time. This structure could be further enriched – for example by using time series models for the development of subject and cluster effects over time, or by using cluster by treatment interaction terms to model variation in treatment effects across clusters. In its current form the model expresses the tension between cluster‐effects and time‐effects for the design of cluster studies in a form susceptible to exact analysis. At the design stage, the correlations are assumed known and, following the strategy of Hussey and Hughes 11, we use the model to compute the precision of unbiased linear estimates of a fixed treatment effect. This approach is moment‐based and makes no explicit distributional assumption, although it is clearly justifiable when the data are normally distributed with the prescribed correlation‐structure. In some other cases, where central limiting arguments can be deployed, it approximates to the best approach – for example, for binary outcomes when *m* is large. The additivity requirement in the model can be relaxed within a generalised linear mixed model, with fixed and random effects on a transformed scale. Then our results may still apply approximately if these effects are relatively small, permitting a linear approximation on the natural scale.

Several aspects of this work offer potential applications in the design of both cross‐sectional cluster studies (where each subject provides a single outcome measurement) and of cohort designs (where repeated measures are taken on the same subjects). First, the expression for precision in terms of the CMC leads to a convenient graphical tool for comparing the performance of competing study designs, and provides a simple approach to the computation of design effects which are useful in sample size calculations. Second, the class of hybrid designs emerge as admissible designs, at least in large studies, in the sense that any design not of this form is (weakly) dominated by at least one design that is. Cross‐sectional studies can often be conceptualised as a series of observations taken over a large number of weeks or months, in which case the hybrid results may be directly applicable. Third, a simple search algorithm is proposed for finding optimal designs when the ‘large‐study’ results are inapplicable. This is often the case in cohort studies, where the number of observation times is limited to the (usually small) number of repeat measures on each subject.

The precise nature of the optimal design depends on the value of the CMC which, in common with the traditional ICC, may be difficult to predict with certainty. An alternative strategy is to look for a design that reduces the possible loss of precision because of an unknown CMC. For this purpose the near‐minimax hybrids of section 3.5 are offered as practical alternatives to more traditional designs.

## Appendix A The precision of the treatment effect estimate (equation 3)

### A.1

We outline a derivation for the precision of the BLUE for *θ* in model (2). Without loss of generality, take σ^2^ = 1.

Consider a transformation of the observation matrix *Y* (with elements *Y_ij_*) given by

$$U^{T}=HY^{T}$$

where *H* is a *T* × *T* orthogonal matrix (i.e. *H^T^H = I_T_*) with first row given by $\frac{1}{\sqrt{T}}\left(1,\text{1},\ldots ,1\right)$. (Throughout we use the conventional notation that the superscript *^T^* denotes matrix transposition. This is not to be confused with *T*, the time‐dimension in the model.) Also define the *K* × *T* matrices *Z* and *η* by *Z* ^*T*^ = *HJ* ^*T*^, *η* ^*T*^ = *Hε* ^*T*^. Then a model for the transformed observations corresponding to (2) is

(A1) $$\begin{array}{l} U_{i1}=\sqrt{T}\gamma_{i}+{\left(Ht\right)}_{1}+\theta Z_{i1}+\eta_{i1};\;i=1,\ldots K.\\ U_{ij}={\left(Ht\right)}_{j}+\theta Z_{ij}+\eta_{ij};\;i=1,\ldots K,\;j=2,\ldots ,T \end{array}$$

where {*η*_*ij*_; 1 ≤ *i* ≤ *K*, 1 ≤ *j* ≤ *T*} is a set of uncorrelated random variables with mean zero and variance (1 − *ρ*). The orthogonality of *H* implies that the elements of the matrix *U* are uncorrelated with one another. Thus, as *j* varies from 1 to *T*, the expressions in (A1) represent a series of *T* linear regression models for *U_ij_* on *Z_ij_*, with intercepts (*Ht*)*_j_* and common slope *θ*. For j = 1, the error variance is $\mathrm{var}\left(\sqrt{T}\gamma_{i}+\eta_{i1}\right)=T\rho +1-\rho$; for 2 ≤ *j* ≤ *T*, the error variance is (1 − *ρ*). The estimation problem for *θ* reduces to that of finding the common slope in a set of uncorrelated regressions. The precision of the pooled estimate for *θ* is just the sum of the precisions of the BLUEs in the separate models. In a simple linear regression model with explanatory variables *x_i_* the precision of the slope estimate is $\sum {\left(x_{i}-\bar{x}\right)}^{2}÷\;\left(\text{Error Variance}\right)$. Hence the precision of the pooled estimate is given by:

(A2) $$\frac{1}{T\rho +1-\rho }\sum_{i=1}^{K}{\left(Z_{i1}-{\bar{Z}}_{•1}\right)}^{2}+\frac{1}{1-\rho }\sum_{j=2}^{T}\sum_{i=1}^{K}{\left(Z_{ij}-{\bar{Z}}_{•j}\right)}^{2}=\frac{1}{1-\rho }\left[-\frac{T\rho }{T\rho +1-\rho }\sum_{i=1}^{K}{\left(Z_{i1}-{\bar{Z}}_{•1}\right)}^{2}+\sum_{j=1}^{T}\sum_{i=1}^{K}{\left(Z_{ij}-{\bar{Z}}_{•j}\right)}^{2}\right]=\frac{1}{1-\rho }\left[\sum_{j=1}^{T}\sum_{i=1}^{K}{\left(Z_{ij}-{\bar{Z}}_{•j}\right)}^{2}-R\sum_{i=1}^{K}{\left(Z_{i1}-{\bar{Z}}_{•1}\right)}^{2}\right]=\frac{1}{1-\rho }\left[\sum_{j=1}^{T}\sum_{i=1}^{K}{\left(J_{ij}-{\bar{J}}_{•j}\right)}^{2}-R\sum_{i=1}^{K}T{\left({\bar{J}}_{i•}-{\bar{J}}_{••}\right)}^{2}\right],\;\left(A2\right)$$

from which (3) follows. The last equality holds because $Z_{i1}=\sqrt{T}{\bar{J}}_{i•}$, and

$$\sum_{j=1}^{T}\sum_{i=1}^{K}{\left(Z_{ij}-{\bar{Z}}_{•j}\right)}^{2}=tr\left[Z^{T}\left(I_{K}-\frac{1}{K}1_{K}1_{K}^{T}\right)Z\right]=tr\left[HJ^{T}\left(I_{K}-\frac{1}{K}1_{K}1_{K}^{T}\right)JH^{T}\right]=tr\left[H^{T}HJ^{T}\left(I_{K}-\frac{1}{K}1_{K}1_{K}^{T}\right)J\right]=tr\left[J^{T}\left(I_{K}-\frac{1}{K}1_{K}1_{K}^{T}\right)J\right]=\sum_{j=1}^{T}\sum_{i=1}^{K}{\left(J_{ij}-{\bar{J}}_{•j}\right)}^{2}\text{.}$$

#### Fixed cluster effects

If the cluster effects γ*_i_* are treated as fixed, the regression model for *U*_*i*1_ in (A1) is not identifiable and does not yield an estimate for *θ*. Its contribution to the precision of the pooled estimate is zero, which is tantamount to setting *R* = 1 in the expression (A2).

#### Time‐effects modelling

The model in (1) has one parameter for each of the *T* observation times in the study. However, the argument can be extended to show that the expression (A2) holds under more restrictive time‐effects models. To be precise, if the *T* × 1 vector of time effects is modelled as *t* = *Xβ* for some design matrix *X*, where β is a vector of unknown parameters, the result (A2) holds provided that the vector *J^T^*1*_K_* (whose jth element is *J*_• *j*_) lies in the column‐space of *X*. In a stepped design this amounts to the requirement that the model is rich enough to include a separate time parameter for any period in which no cluster experiences an uptake event. For the designs in Figure 1, it means that (A2) remains true provided the model includes at least one time‐parameter for each of the columns depicted in the corresponding figure.

## Appendix B Design coefficients for standard designs (Table 1)

### B.1

As indicated in section 2, the design coefficients, *a_D_* and *b_D_* may be interpreted in terms of variances generated by a uniform probability measure on the lattice points {(*i*, *j*); 1 ≤ *i* ≤ *K*, 1 ≤ *j* ≤ *T*}. Under this probability measure we have *a*_*D*_ = *E*_*j*_ var_*i*_(*J*_*ij*_|*j*)≡ var_*ij*_ *J*_*ij*_ − var_*j*_ *E*_*i*_(*J*_*ij*_|*j*) and $b_{D}={\mathrm{var}}_{i}{\bar{J}}_{i•}={\mathrm{var}}_{i}E_{j}\left(J_{ij}|i\right)\text{≡}{\mathrm{var}}_{ij}J_{ij}-E_{i}{\mathrm{var}}_{j}\left(J_{ij}|i\right)$.

CXO: The row (i.e. cluster) mean ${\bar{J}}_{i•}=\frac{1}{2}$, a constant for all *i*, and, under the uniform measure on the lattice points, the conditional distribution of *J_ij_* for fixed *j* is Binomial(1, ½). So, for this design, $a_{BCO}=\frac{1}{4}$ and *b*_*BCO*_ = 0.

PD: Here the distribution of ${\bar{J}}_{i•}$ is Binomial(1, ½), as is the conditional distribution of *J_ij_* for fixed *j*. Hence $a_{PD}=b_{PD}=\frac{1}{4}$.

The following result is useful for calculating coefficients for stepped‐wedge and DCD‐designs.

#### Lemma

Suppose that *a_D_* and *b_D_* are the design coefficients for a design *D* with layout matrix *J* containing *n_J_* columns. Let *D*′ be the design obtained when *J* is augmented to include *n*_0_ additional columns of zeros and *n*_1_ additional columns of 1 s. This amounts to adding baseline observations and post‐implementation observations in all clusters. Then the design coefficients for *D*′ are given by *a*_*D*′_ = *qa*_*D*_, *b*_*D*′_ = *q* ^2^ *b*_*D*_ where $q=\frac{n_{J}}{n_{J}+n_{0}+n_{1}}$ is the proportion of the columns in the new layout occupied by the original matrix *J.*

To prove this, note that the row means under *D*′ are given by $\bar{J}\prime_{i•}=\frac{n_{1}+n_{J}{\bar{J}}_{i•}}{n_{J}+n_{0}+n_{1}}$, with variance $q^{2}{\mathrm{var}}_{i}{\bar{J}}_{i•}=q^{2}b_{D}$. Also the variances within the added columns are zero, so that *a*_*D*′_ = *E*_*j*_ var_*i*_(*J*′ _*ij*_|*j*) = (1 − *q*) × 0 + *qE*_*j*_ var_*i*_(*J*_*ij*_|*j*) = *qa*_*D*_ as required.

DCD_*p*,*q*,*r*_: A straightforward application of the lemma to the case where the (unaugmented) base design is parallel (PD) shows that $a_{DCD}=\frac{q}{4},b_{DCD}=\frac{q^{2}}{4}$.

An important consequence of the lemma is that the design coefficients for any design are unaffected when initial baseline observation periods are exchanged for post‐implementation periods and vice versa. In either case they depend only on the proportion, *q*, of time‐points devoted to the central part of the design.

#### Stepped‐wedge designs

MSW*_g_*: Consider a truncated version of the SW*_g_* design in which the first column of control observations is absent. If there is just one cluster in each group, the corresponding *J*‐matrix is square. For example, when *g* = 4, $J=\left[\begin{array}{cccc} 0 & 0 & 0 & 1\\ 0 & 0 & 1 & 1\\ 0 & 1 & 1 & 1\\ 1 & 1 & 1 & 1 \end{array}\right]$. In this layout the proportion of post‐implementation periods is $\frac{1}{g}$ and there are no baseline periods. Hence, from the lemma, the design coefficients will be the same as those for the corresponding MSW design in which the baseline and post‐implementation proportions are both equal to $\frac{1}{2g}$. In the truncated layout, the row and column means are identical, and uniformly distributed on the set $\left\{\frac{1}{g},\frac{2}{g},\ldots ,\frac{g-1}{g}\;,1\right\}$. This distribution has variance $\frac{1}{12}\left(1-\frac{1}{g^{2}}\right)=b_{MSW}$. The proportion of 1s in *J* is $\frac{1}{2}\left(1+\frac{1}{g}\right)$ so that ${\mathrm{var}}_{ij}J_{ij}=\frac{1}{4}\left(1-\frac{1}{g^{2}}\right)$. Hence $a_{MSW}=\frac{1}{4}\left(1-\frac{1}{g^{2}}\right)-\frac{1}{12}\left(1-\frac{1}{g^{2}}\right)=\frac{1}{6}\left(1-\frac{1}{g^{2}}\right)$.

SW: The coefficients for SW*_g_* can be obtained in a similar way. Here an extra baseline period is added to the truncated design. Using the lemma, the design coefficients are given by:

$$a_{SW}=\frac{g}{g+1}a_{MSW}=\frac{g}{g+1}\cdot \frac{1}{6}\left(1-\frac{1}{g^{2}}\right)=\frac{1}{6}\left(1-\frac{1}{g}\right)$$

and

$$b_{SW}={\left(\frac{g}{g+1}\right)}^{2}b_{MSW}={\left(\frac{g}{g+1}\right)}^{2}\cdot \frac{1}{12}\left(1-\frac{1}{g^{2}}\right)=\frac{1}{12}\left(1-\frac{2}{g+1}\right)\text{.}$$

#### Transposed layouts and hybrid designs

Consider the design layout *J^T^* obtained by interchanging the rows and columns of *J*. The design coefficients for the transposed layout can be expressed as $a_{D^{T}}=\pi \left(1-\pi \right)-b_{D}$ and $b_{D^{T}}=\pi \left(1-\pi \right)-a_{D}$ where π is the proportion of 1 s in the layout, using the fact that var *J*_*ij*_ = *π*(1 − *π*).

The layout for a hybrid design, *^β^*H_*g*_, can be written as

Here *O* and *U* represent matrices of 0 s and 1 s respectively, and *J_MSW_* is the layout for a MSW*_g_* design. Using the MSW coefficients (in conjunction with the result of the lemma) the hybrid design coefficients are obtained as $a_{H}=\frac{1}{4}-\frac{\beta^{2}}{12}\left(1+\frac{2}{g^{2}}\right)$ and $b_{H}=\frac{1}{4}-\frac{\beta }{12}\left(2+\frac{1}{g^{2}}\right)$.

## Appendix C Derivation of equation (6)

### C.1

First note that the quantities *a_D_* and *b_D_* that feature in (3) can be expressed as:

$$\begin{array}{l} TKa_{D}=\sum_{i=1}^{K}\sum_{j=1}^{T}J_{ij}^{2}-\frac{1}{K}\sum_{j=1}^{T}J_{•j}^{2}=J_{••}-\frac{1}{K}\sum_{j=1}^{T}J_{•j}^{2},\;\left(\text{since}\;J_{ij}^{2}\text{≡}J_{ij}\right);\\ TKb_{D}=\frac{1}{T}\sum_{i=1}^{K}J_{i•}^{2}-\frac{1}{TK}J_{••}^{2} \end{array}$$

Now assume that the clusters are labelled according to the order in which they receive the intervention. Then, for any value of *j*, we have *J_ij_* = 1 only for the first *J*_•*j*_ clusters, where $J_{•j}=\sum_{i=1}^{K}J_{ij}$ is the total number of clusters that have received the intervention by time j. Similarly, for each value of *i*, we have *J_ij_* = 1 only for the last *J*_*i*•_ time‐points. It follows that $\sum_{i=1}^{K}iJ_{ij}=\sum_{i=0}^{J_{•j}}i=\frac{1}{2}J_{•j}\left(J_{•j}+1\right)$ and $\sum_{j=1}^{T}jJ_{ij}=\sum_{j=T-J_{i•}+1}^{T}i=\frac{1}{2}J_{i•}\left(2T-J_{i•}+1\right)$. (For the second expression we observe the convention that a sum is null if the lower limit exceeds the upper limit. This delivers the correct result when *J*_*i*•_ = 0.)

Using these expressions we find that

$$2\sum_{i=1}^{K}\sum_{j=1}^{T}y_{i}J_{ij}=\frac{2}{K}\sum_{i=1}^{K}\sum_{j=1}^{T}\left(i-\frac{K+1}{2}\right)J_{ij}=\frac{1}{K}\sum_{j=1}^{T}J_{•j}\left(J_{•j}+1\right)-\left(1+\frac{1}{K}\right)J_{••}=\frac{1}{K}\sum_{j=1}^{T}J_{•j}^{2}-J_{••}=-TKa_{D}\text{,}$$

and

$$2\sum_{i=1}^{K}\sum_{j=1}^{T}x_{j}J_{ij}=\frac{2}{T}\sum_{i=1}^{K}\sum_{j=1}^{T}\left(j-\frac{T+1}{2}\right)J_{ij}=\frac{1}{T}\sum_{i=1}^{K}J_{i•}\left(2T-J_{i•}+1\right)-\left(1+\frac{1}{T}\right)J_{••}=J_{••}-\frac{1}{T}\sum_{i=1}^{K}J_{i•}^{2}=TKb_{D}+J_{••}-\frac{1}{TK}J_{••}^{2}\text{.}$$

Thus $TK\left(a_{D}-Rb_{D}\right)=2\sum_{i,j}\left(Rx_{j}-y_{i}\right)J_{ij}-R\left(J_{••}-\frac{1}{TK}J_{••}^{2}\right)$ which, on substituting in (3), gives the desired result.

## Appendix D Unconstrained optimal design of large stepped studies

### D.1

It is shown in section 3.1 that the optimal uptake boundary corresponds to a line segment of slope *R* in the *x–y* plane of the form *y – Rx* = *η*, for some value of *η*. Let *A*(*η*) be the area of the region in the unit square below the line segment – i.e. the region occupied by the treated units. Then *J*_• •_ ~ *TKA*(*η*) and

$${\left(TK\right)}^{-1}\left\{2\sum_{i,j}\left(Rx_{j}-y_{i}\right)J_{ij}-R\left(J_{••}-\frac{1}{TK}J_{••}^{2}\right)\right\}+O\left(\min{\left(T,K\right)}^{-1}\right)=-2\int_{-\frac{1}{2}\left(1+R\right)}^{\eta }\eta \prime dA\left(\eta \prime \right)-RA\left(\eta \right)\left(1-A\left(\eta \right)\right)\text{.}$$

The right‐hand side has a turning value when the derivative with respect to *η* is zero. I.e. when− 2*ηA*′ (*η*) − *RA*′ (*η*) + 2*RA*(*η*)*A*′ (*η*) = 0, where $A\prime \left(\eta \right)=\frac{dA\left(\eta \right)}{d\eta }>0$.

Dividing through by *A*′(*η*) we find that the maximum precision is obtained when

$$\eta =\left(A\left(\eta \right)-\frac{1}{2}\right)R\text{.}$$

This equation is clearly satisfied when *η* = 0 because *A*(0) = ½, and the solution corresponds to a balanced design. Detailed consideration of the functional form of *A*(*η*) – which is straightforward but not given here – reveals that the equation has no other root. Thus the optimal design is approximately balanced when *T* and *K* are large, as claimed in section 3.4.
